# High insertion torque versus regular insertion torque: early crestal bone changes on dental implants in relation to primary stability—a retrospective clinical study

**DOI:** 10.1186/s40729-024-00540-3

**Published:** 2024-05-03

**Authors:** Jörg-Martin Ruppin, Michael Stimmelmayr

**Affiliations:** 1 Penzberg, Germany; 2 Department of Prosthetic Dentistry, University Hospital, LMU Munich, Munich, Germany; 3 Cham, Germany

**Keywords:** Bone density, Bone loss, Immediate loading, Insertion torque, Primary stability, RFA analysis

## Abstract

The aim of the presented retrospective study was to evaluate the early crestal bone changes around an implant type designed for high primary stability. A total number of 111 implants placed clinically were evaluated regarding insertion torque, bone density, implant stability quotient (ISQ) and early crestal bone loss from standardized digital radiographs. The implants were allocated in two groups: the „regular torque “ group contained all implants that achieved less than 50 Ncm as final insertion torque (n = 63) and the „high torque“ group contained the implants that achieved 50–80 Ncm (n = 48). To avoid possible damage either to the implant´s inner connection or to the bone by application of excessive force, a limit of 80 Ncm was set for all surgeries. All implants underwent submerged healing for three months. ISQ measurements and standardized digital radiographs were taken at day of insertion and at day of second stage surgery. The bone loss was measured on the mesial and distal aspect of the implant. The data evaluation showed the following results: Mean bone loss was 0.27 ± 0.30 mm for the high torque group and 0.24 ± 0.27 mm for the regular torque group. The difference was not statistically significant (p = 0.552). In the two groups, no complications nor implant loss occurred. For the evaluated implant type, there was no significant difference in crestal bone changes and complication rate between high and regular insertion torque in the early healing period.

**Graphical Abstract:**

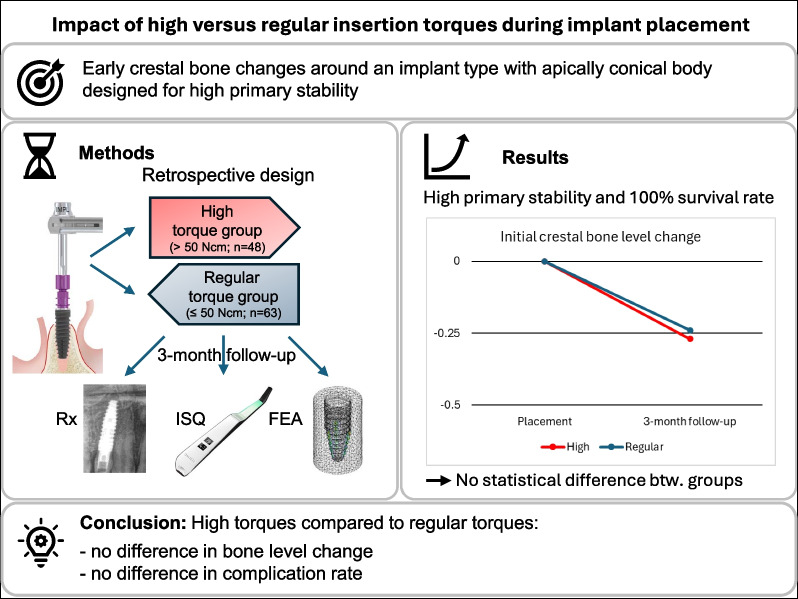

## Background

In the recent research, a lot of emphasis is drawn to the question of immediate implant placement and immediate loading [3, 4, 19, 22, 23]. The most important factor for successful immediate loading is a sufficient primary stability [15], as the implant stability will be reduced during the healing due to resorptive processes in the healing bone before reaching the final stability after 8–12 weeks [19, 21, 23]. If the primary stability is insufficient, there will be a higher risk for implant loss due to failing osseointegration [8].

There is evidence suggesting a close correlation between insertion torque values (ITV) and the pressure that is applied to the surrounding bone by the dental implant [21]. Excessive pressure around an implant as may be caused by high ITVs can damage the bone—especially the cortical crestal bone—by causing microfractures and cellular apoptosis, thus leading to excessive resorption and remodeling of the healing bone [9, 26]. Due to the different mechanical stiffness, different bone densities may show different pressure distribution around an implant [17].

Until today, the literature is inconsistent with the question whether using high ITVs will cause higher marginal bone loss or higher implant failure rates [4, 5, 9, 12, 13, 18, 26]. In the presented study, the implants were allocated in two groups: the „regular torque“ group and the „high torque“ group. Multiple studies have shown that insertion torques between 15 and 45 Ncm are sufficient to allow primary stability and successful osseointegration [4, 7]. Modern implant types with apically tapered bodies are designed to achieve high primary stability with torques above 35Ncm. Therefore, and based on the systematic reviews by Lemos et al. and Atieh et al. [2, 18] as well as a randomized clinical study by Alfonsi et al. [1], the dividing criteria between high and regular torque groups was defined as 50 Ncm.

The aim of the presented retrospective study was to evaluate the early crestal bone loss (EBL) during a healing period of three months after implant placement and to compare it to type of bone (Type 1–4) and the measured insertion torque.

## Methods

The study was approved by the ethics committee of the Bayerische Landesärztekammer (Ref. 2022-1135) and the patient data was anonymized for the analysis. The study was registered in a study registry (Deutsches Register für Klinische Studien Ref DRKS00029995).

From the patients treated in a private dental practice by one skilled professional surgeon (JMR) in the period from January 2021 to June 2022 all implants fulfilling the inclusion criteria were included in the evaluation. The patients had to be more than 18 years of age and treated with one or more implants (CAMLOG PROGRESSIVE-LINE, Camlog, Wimsheim, Germany). Exclusion criteria were history of systemic diseases that would contraindicate oral surgery, long-term nonsteroidal anti-inflammatory drug therapy, intravenous bisphosphonate therapy, and untreated periodontal disease. The surgical procedure was identical for all cases: late implant placement (> 3 months healing period after tooth extraction) with unloaded, submerged implant healing for three months. Included were only implants without augmentation and implants with simultaneous sinus lift (vertical approach), but no crestal augmentation techniques. Implant bed preparation and insertion of the implants were performed according to the manufacturer´s specifications. Implant length varied from 9 to 13 mm, implant diameter varied from 3.3 to 5.0 mm. All patients received oral antibiotics for six days postop (Clindamycin 600 mg twice a day). Sutures were removed after 10 days.

Two patients (2 implants) who had experienced wound dehiscence at the implant site resulting in a prolonged healing time were excluded in order to keep the data and follow-up time consistent. Therefore, data of a total of 63 patients (111 implants) were retrospectively extracted from the clinical data including risk factors such as smoking or diabetes. According to the treatment protocol of the dental practice, the insertion torque value (ITV) was registered digitally using an implant motor with integrated real-time torque measurement (iChiropro, Bien Air Deutschland GmbH, Germany). At day of implant placement and of second stage surgery RFA measurements of the implants were taken and thus the implant stability quotient (ISQ) was measured (Osstell Beacon, W&H Dental Group, Austria). Furthermore, the bone density referring to the Lekholm and Zarb classification [17] was evaluated. All data were stored in the OsstellConnect database software.

For every patient, an individual X-ray holder for periapical radiographs was used (Rinn XCP Bite Block, Rinn XCP Film Holder, DentsplyRinn, USA) that was modified using bite registration material (R–SI Metal Bite Blue, R-Dentalerzeugnisse GmbH, Germany), thus enabling the taking of digital radiographs (Vista Scan, Dürr Dental SE, Germany) with an identical projection at day of implant placement and at day of second stage surgery. The images were digitally superimposed and the difference between the bone level and the implant shoulder at the mesial and distal aspect of the implants was measured using a dental radiograph imaging software (byzz next digital radiograph software, Orange Dental, Germany). For evaluation, the mean of the mesial and distal measurements was taken.

The implant data were divided into two groups: the „regular torque“ group contained all the implants that had an ITV of below 50 Ncm, whereas the „high torque“ group contained implants with an ITV between 50 and 80 Ncm.

The study data were descriptively analyzed using IBM SPSS V25.0 (IBM Corp., Armonk, NY, USA). Due to the fact that there was no normal distribution in the bone level data as well as in the ISQ and ITV data, non-parametric tests were used to compare the means of these parameters between the two groups “regular torque” versus “high torque” (significance level at p < 0.05). A power analysis considering the bone level results of an equivalent designed study by Marconcini et al. [20] delivered a minimal sample size of n = 23 (power 0.8 and significance level p < 0.05).

To determine the stress distribution resulting from the threading of the dental implant in the jaw, a Finite Element Method (FEM) simulation was constructed using a FEM simulation software (Catia V5, Dassault Systèmes, France) within the "Generative Structural Analysis" environment. To represent the jaw as a dummy structure, a generic cylinder was created. In order to account for the missing volume due to the borehole, the geometry of the drill was rotated about its central axis, and the resulting bore volume was subtracted from the volume of the cylindrical jaw dummy. Subsequently, the implant was placed in its target position as specified by the manufacturer. The volume of the implant component in this position was also subtracted from the volume of the cylindrical jaw dummy. For the simulation's boundary conditions, the jaw was modeled as being rigidly fixed, while the implant was configured with one degree of freedom, allowing rotation about its central axis within the jaw. The application of force in the final position of the implant was simulated through the milling groove. For this purpose, a contact surface was defined at the outer edge of the milling groove, representing the interaction between the implant and the jaw. Torques ranging from 20 to 80 Ncm were modeled as loads on the implant, acting about the rotational axis of the implant. The material properties of the jaw were modeled as isotropic with a Young's Modulus of 400 MPa. For the implant, titanium with a Young's Modulus of 114,000 MPa was used.

## Results

**Fig. 1:**
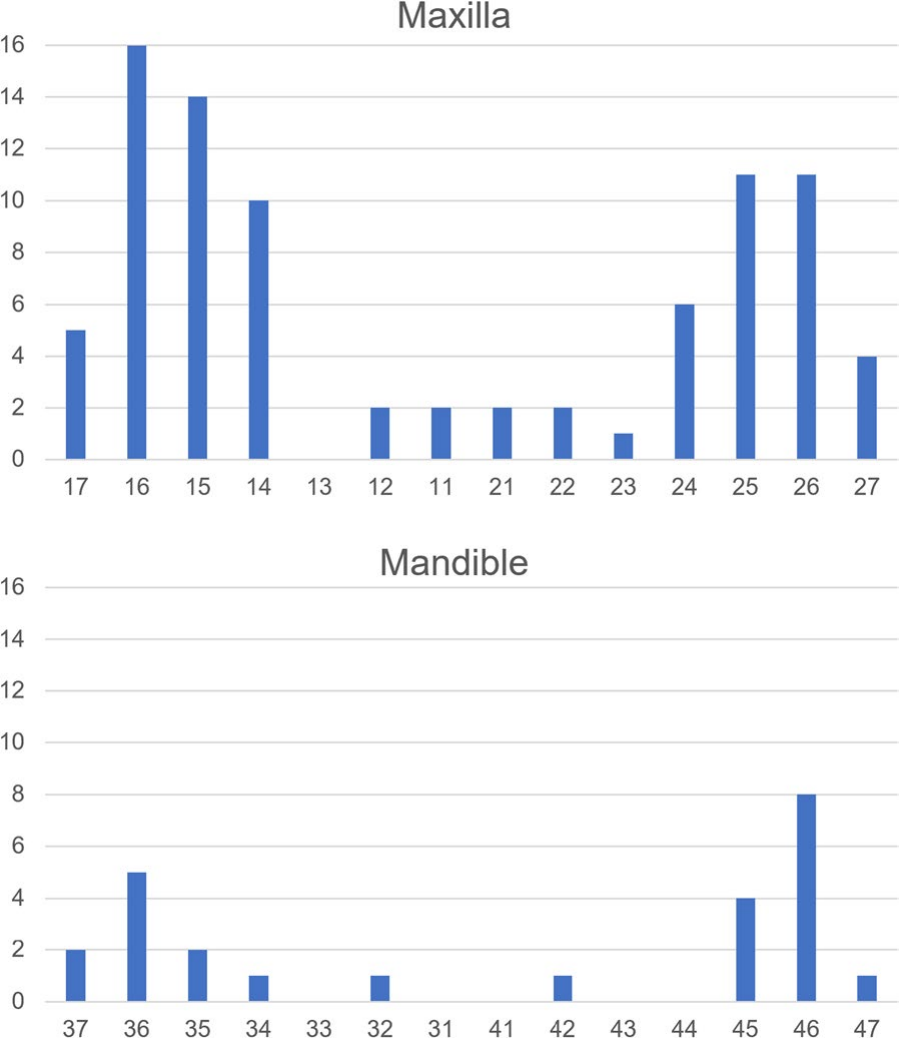
Distribution of implants in maxilla and mandible. Number of implants placed according to the FDI tooth numbering system

In total, 111 implants inserted in 63 patients in the period between January 2021 and February 2022 were evaluated. Approximately two third were female (n = 41) and one third were male (n = 22), age ranging from 23 to 84 years. The patients’ medical history included risk factors like smoking (15%), diabetes (2%), cardiovascular disease (24%), penicillin allergy (9%), and autoimmune disease (2%). The remaining 48% of the population were assessed with no risks. Eighty-six implants were placed in the maxilla (77.5%) and 25 in the mandible (22.5%). The distribution of implant position is shown in Fig. 1.

After a mean healing of 3.3 ± 0.3 months (max. 4.2, min 2.8), 83 implants were restored with single crowns, 24 implants with partial dentures, and 4 implants with full-arch restoration. The distribution of the bone quality assessed according to Lekholm and Zarb [17] at the implant bed preparation is given in Table 1.

**Table 1:** Bone quality distribution

Bone quality	Frequency	Percent
1	8	7.2
2	25	22.5
3	42	37.8
4	36	32.4
Total	111	100.0

The implants were allocated into two groups based on the ITV at implant placement: 48 implants (43.2%) achieved an ITV ≥ 50 Ncm, 63 implants (56.8%) were inserted with an ITV less than 50 Ncm. Generally, all implants achieved primary stability. These two groups were taken for further analyses of the influence of high versus regular torques. The ITV and mean ISQ values at day of implant placement for the group “high torque” and “regular torque” as well as for each bone quality are given in Table 2.

**Table 2:** ITV and ISQ at day of implant placement

	N	ITV [Ncm]	ISQ
Total group	111	47.3 ± 18.1	73.7 ± 6.2
High torque group (≥ 50 Ncm)	48	65.8 ± 7.0	76.6 ± 5.2
Regular torque group (< 50 Ncm)	63	33.1 ± 8.6	71.4 ± 6.1
Implants in bone quality 1	8	66.4 ± 8.7	76.1 ± 7.9
Implants in bone quality 2	25	62.7 ± 11.2	76.8 ± 5.6
Implants in bone quality 3	42	46.7 ± 16.4	73.6 ± 5.0
Implants in bone quality 4	36	33.0 ± 12.4	71.0 ± 6.5

ITV and ISQ distribution in the study groups and different bone qualities

### ISQ and ITV evaluated in comparison to bone density

**Fig. 2:**
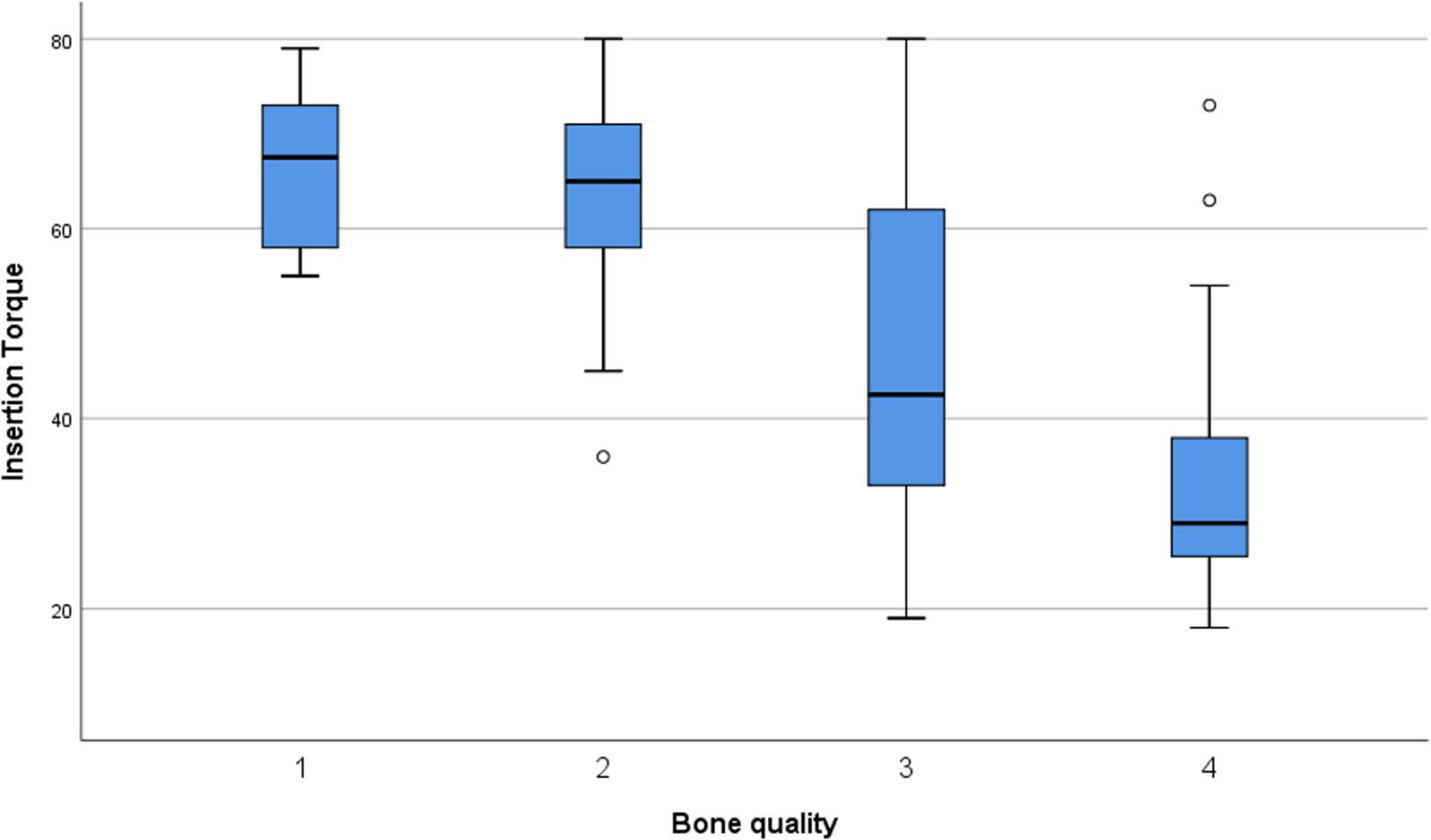
ITV related to bone quality. Box plot diagram of insertion torque (ITV) for each bone quality (1–4)

**Fig. 3:**
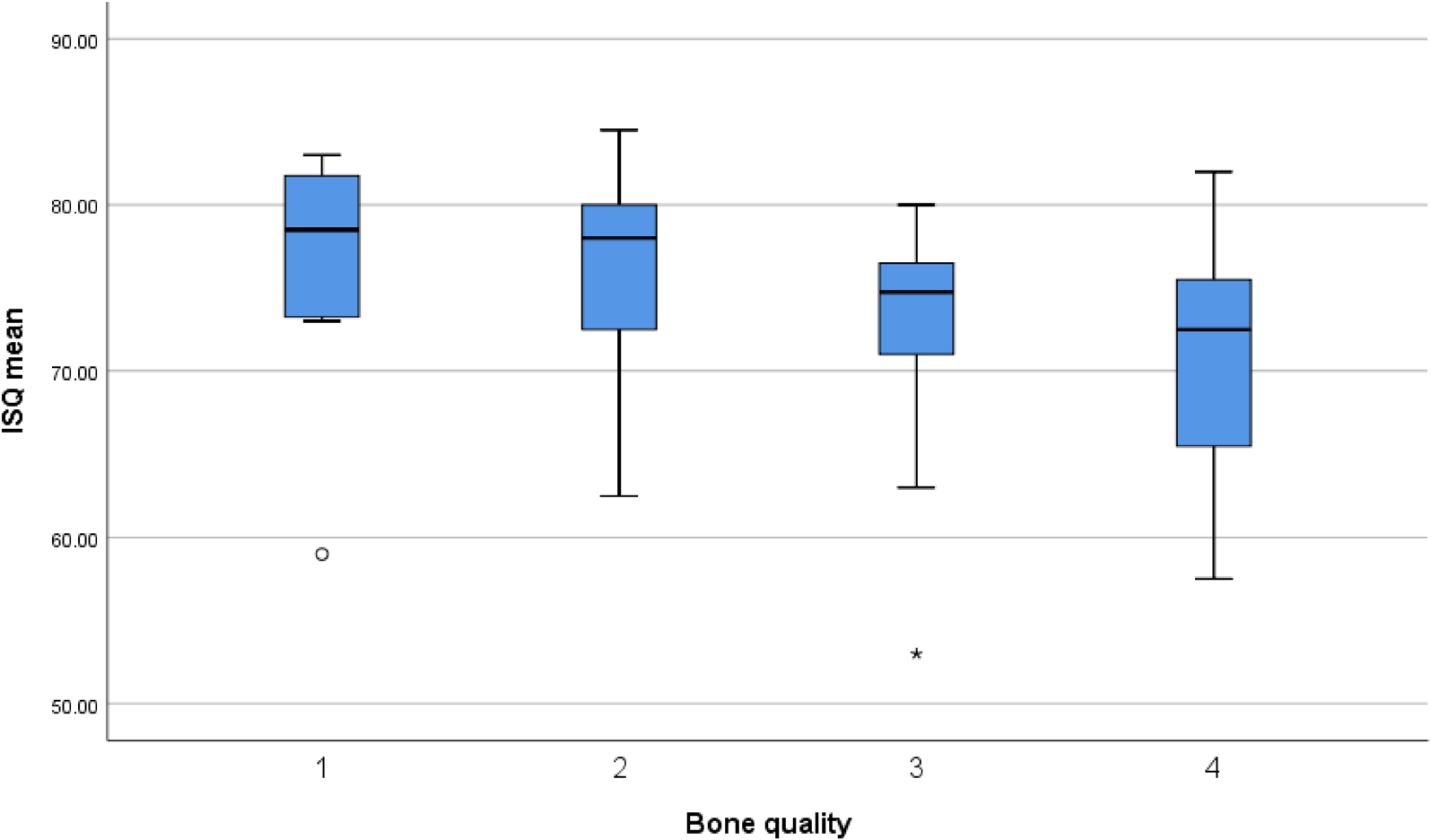
ISQ related to bone quality. Box plot diagram of ISQ at day of implant placement for each bone quality (1–4)

The mean ITV showed highly significant differences between the different bone densities. Only the values within the bone density type 1 and 2 showed no significant difference using the pairwise comparison (Fig. 2). The mean ISQ at implant placement evaluated for each bone density on the other hand showed a statistically significant difference between the values for bone density type 2 and 4. The pairwise comparison revealed no further statistical differences after Bonferroni correction (Fig. 3). Additionally, the ISQ and ITV were correlated to respective bone density classes. Both parameters revealed a weak to moderate correlation to bone density (Correlation coefficient 0.381 (ISQ) and 0.661 (ITV)): High ISQ and ITV with bone densities 1, 2 and lower ISQ and ITV values with bone densities 3, 4.

### ISQ development evaluated over time

The ISQ measurements at re-entry/loading revealed an increase to 78.8 ± 4.7 which was statistically significant (p = 0.000) from the initial measurement at day of implant placement. This finding was also true for the groups high versus regular torque: High-torque: increase of ISQ from 76.6 ± 5.2 to 80.8 ± 4.2; regular-torque: increase of ISQ from 71.4 ± 6.1 to 77.3 ± 4.5. These values suggest a full osseointegration of all implants.

### ITV and ISQ in correlation to each other

**Fig. 4:**
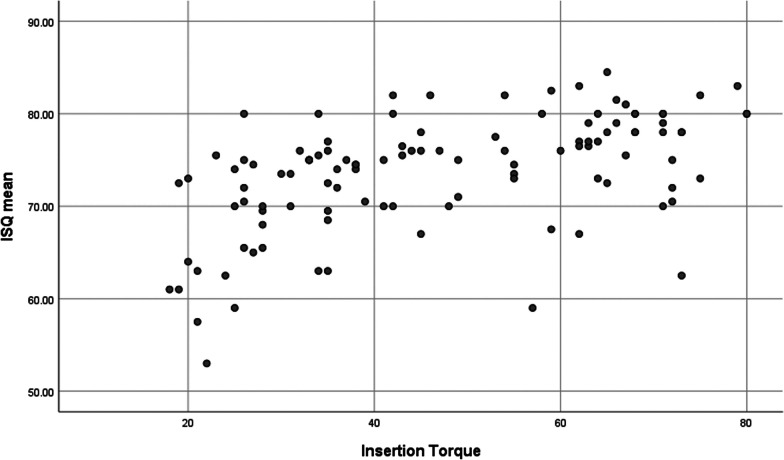
ISQ correlated against ITV. Scatterplot diagram showing the correlation of ISQ and ITV

Considering all implants, a positive moderate linear association was found between the ITV and ISQ at day of implant placement (Pearson correlation: 0.530, p = 0.000). However, in the “high torque” group, no linear correlation between the two parameters was detected (0.149, p = 0.313), contrary to the “regular torque” group, where the Pearson correlation factor indicated a moderate linear association (0.523, p = 0.000) (Fig. 4).

### Marginal bone level change

**Fig. 5:**
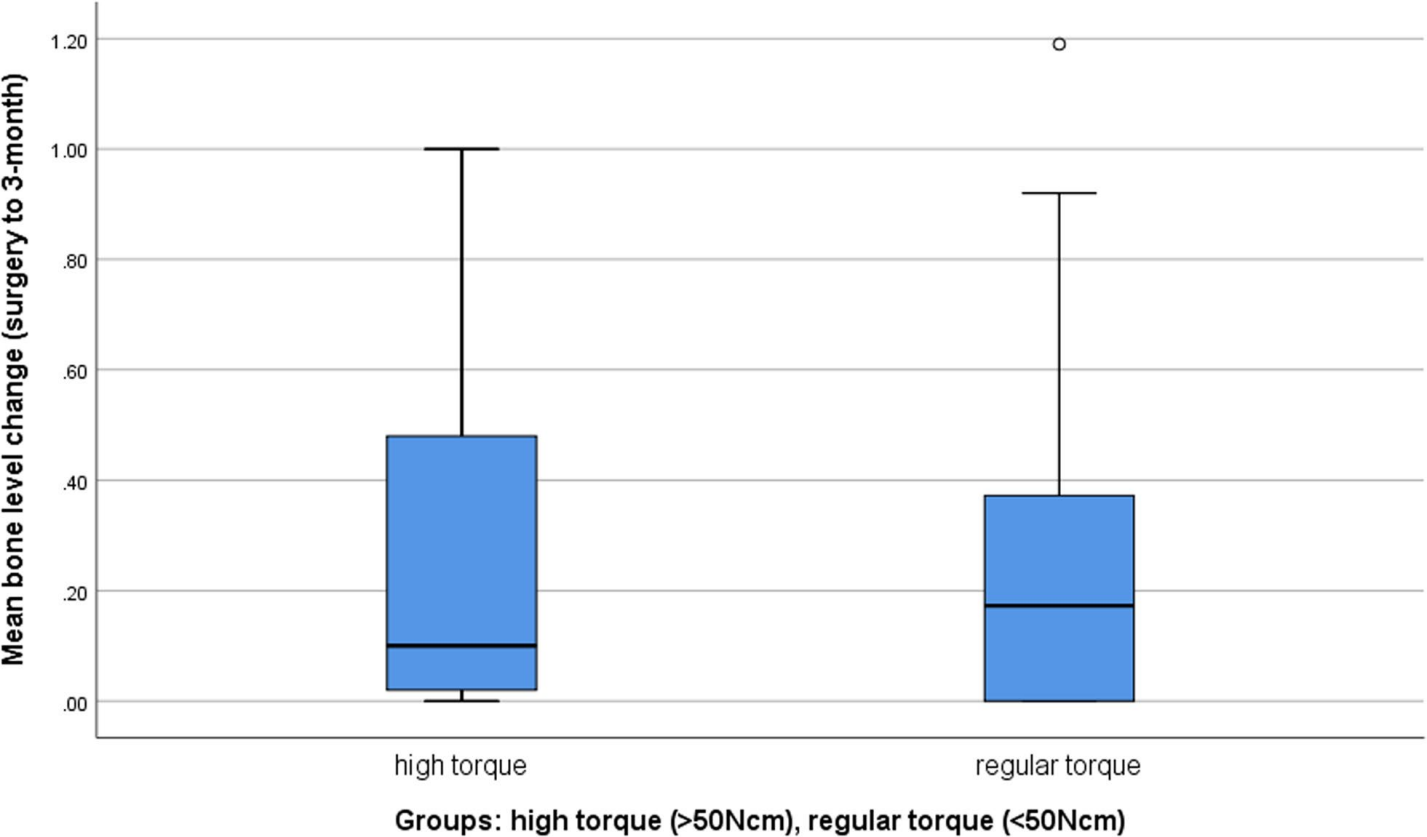
Bone level changes (BLC) in high and regular torque group. BLC between day of implant placement and second stage surgery (in mm)

Radiographs were available at the time of surgery and re-entry for all patients. The total mean marginal bone level loss (MBL) between surgery and re-entry (3-month healing) was 0.25 ± 0.28 mm. Comparing the two groups of insertion torque, the implants with a high insertion torque were measured with a MBL of 0.27 ± 0.30 mm while the implants with regular torque were assessed with a MBL of 0.24 ± 0.27 mm without statistical significance (p = 0.552) (Fig. 5). Also, no correlation between MBL and ISQ or ITV could be detected (Pearson correlation 0.064 and 0.059 respectively).

The BLC for each bone density group (1–4) is given in Table 3. No significant differences in bone level change between the different bone densities were found using a Kruskal–Wallis-Test (p = 0.815).

**Table 3:** BLC in relation to bone quality (in mm)

Bone quality	N	Min (mm)	Max (mm)	Mean (mm)	SD	p
1	8	0.00	0.77	0.25	0.27	0.815
2	25	0.00	0.96	0.25	0.31	
3	42	0.00	1.19	0.26	0.27	
4	36	0.00	1.00	0.24	0.28	
Total	111	0.00	1.19	0.25	0.28	

### Complications

In the observation period, none of the implants experienced any surgical complications and none of the implants was lost after placement.

### Finite elements method

**Fig. 6:**
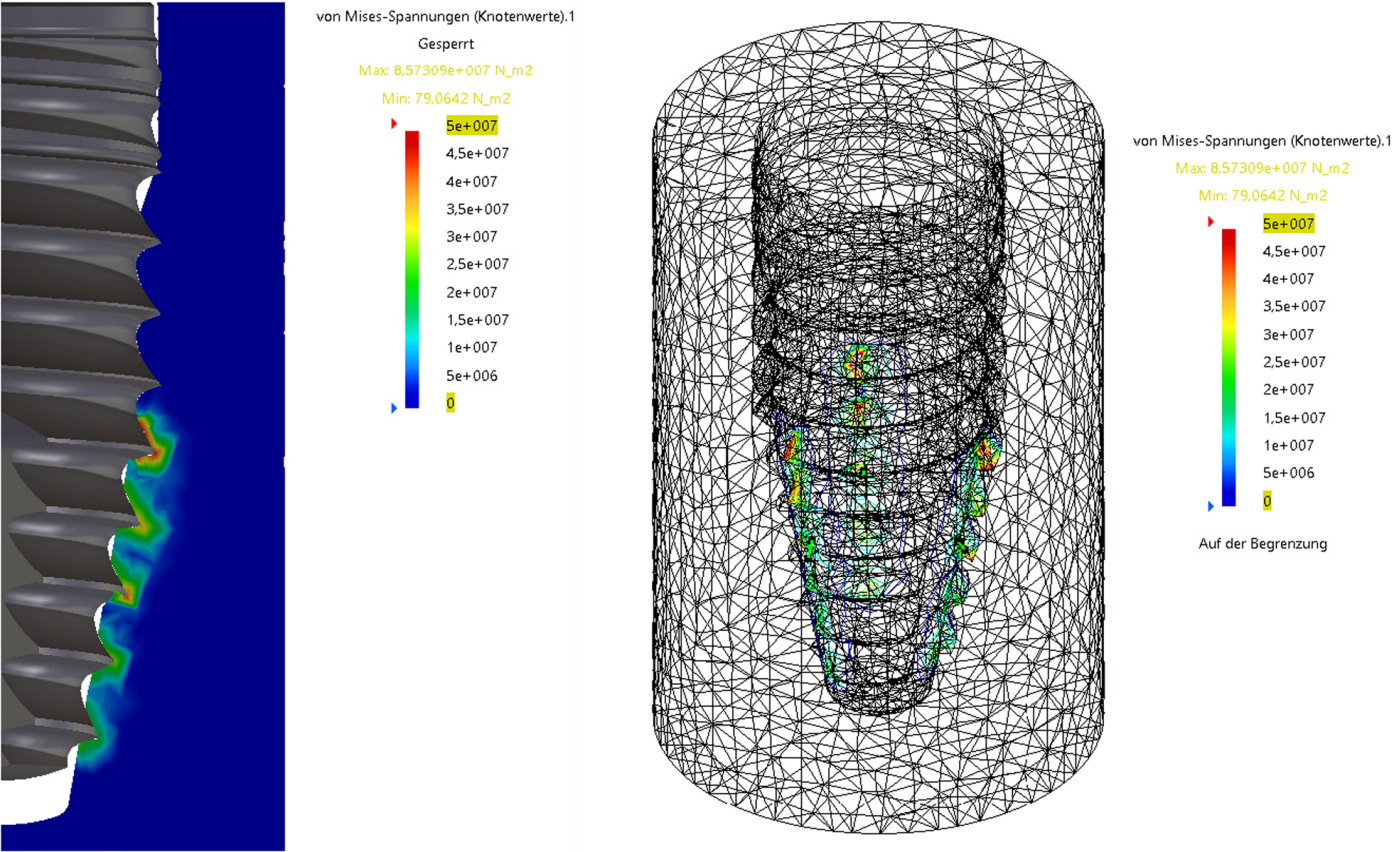
Finite element analysis of the stress at the bone-to-implant surface at ITV of 20 Ncm

**Fig. 7:**
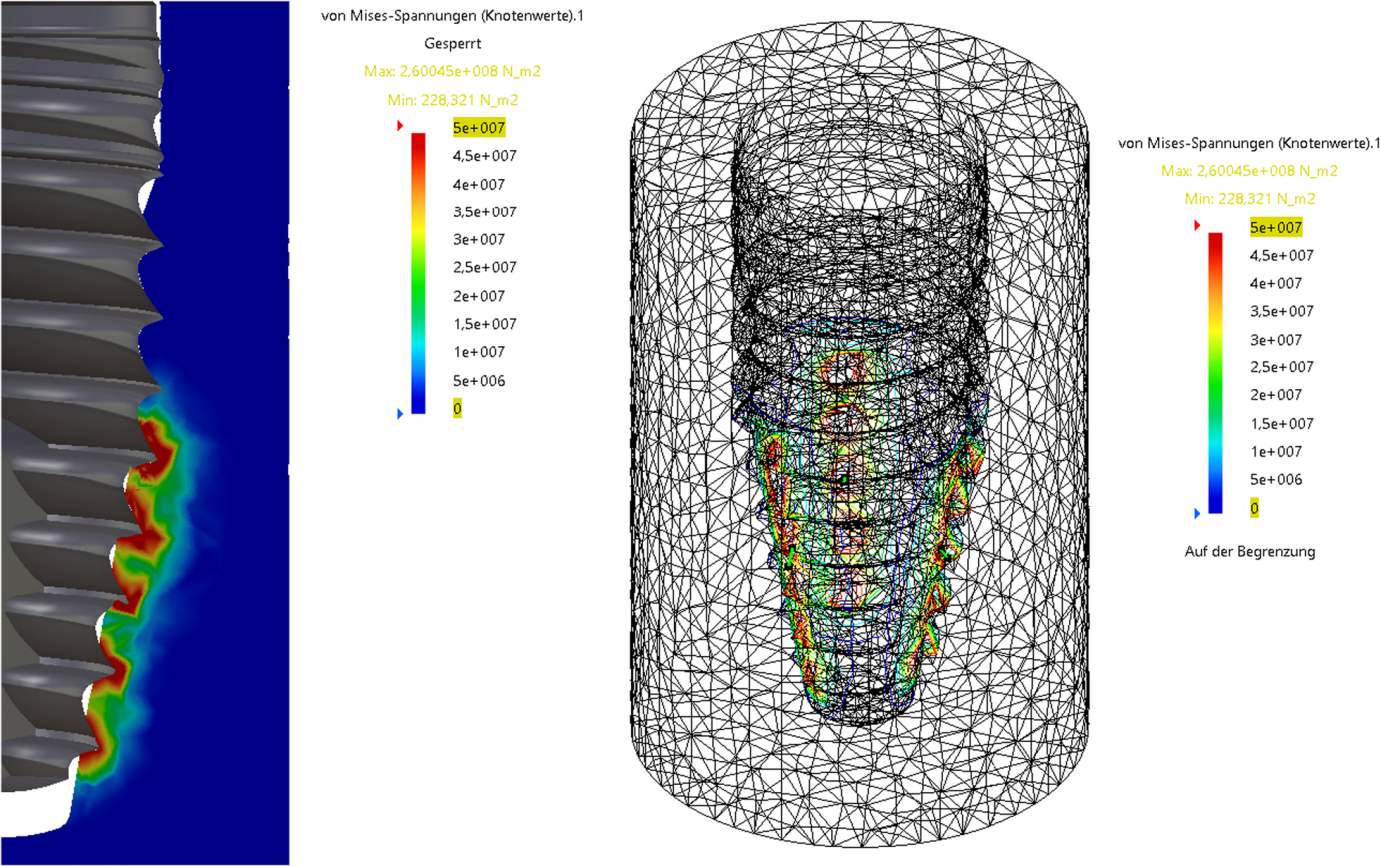
Finite element analysis of the stress at the bone-to-implant surface at ITV of 60 Ncm

The finite element simulation with the herein used implants with the apically tapered geometry indicates that the highest pressure rates are occurring at the implant apex, whereas the crestal part of the implant distributes only minor forces to the surrounding bone, regardless of the insertion torque used (Figs. 6 and 7).

## Discussion

In this retrospective study, the bone level changes related to different insertion torque levels of dental implants were investigated. The measurements of bone level changes were taken at day of second stage surgery, 12–15 weeks after placement. At that point, the bony healing is completed and due to the submerged healing, any other influences than the surgical trauma due to implant placement itself could be excluded. Bone level changes at 3–12 months after implant placement was defined as early bone loss (EBL) by several authors and found to be crucial for long term implant survival [11, 27, 29]. The results in the presented study indicate that the mean marginal bone loss was minimal within the first three months of healing (mean EBL 0.25 ± 0.28 mm) and no significant differences could be observed between the high and low ITV and bone quality 1–4; therefore, the null hypothesis cannot be rejected.

Complete bony healing after tooth extraction takes 8–12 weeks; up to 90% of the crestal bone changes and bone resorption occurs in the first 12 weeks post extractionem and the same principles apply to bony healing after placing implants [10, 25]. For this reason, in the presented study implants were placed in cases with more than 12 weeks post extractionem and the implants healed 12–15 weeks before second stage surgery. The relationship between EBL exposing implant threads and consequently the risk of development of periimplantitis is a well-documented problem [11, 24, 27]. A recent 10-year prospective cohort study including some 1400 implants suggests that EBL is a predictor for long-term peri implant pathology, with a significant higher risk for periimplantitis when EBL exceeds the thresholds of 0.5 mm and 1 mm [29]. In the presented study, no significant differences in EBL were found neither regarding ITV nor ISQ and the mean EBL detected was below the thresholds defined as critical by Windael et al. [29]. These results are of major importance regarding the future implant survival and highlights the necessity of comprehending the precise factors contributing to primary stability, including its mechanism of attainment.

Primary stability of a dental implant is a prerequisite for immediate loading. Any micromotion of the implant beyond a certain threshold can impede osseointegration and ultimately result in implant failure [15].

There is a tendency for using high ITV in order to achieve high primary stability in current literature [13, 18]. From a clinical point of view, it has been made clear that high primary stability is mandatory for immediate loading, especially for single units [6, 14, 16, 26]. Carr et al. [8] have found an increased risk for clinical failure when using an ITV of less than 30 Ncm and two meta-analysis studies have found no indications for higher bone loss or complication rates when using high ITVs [5, 12].

On the other hand, there is strong evidence that excessive intraosseous tissue pressure around an implant as caused by high ITVs can induce bone damage through microfractures and cellular apoptosis. This process can trigger excessive resorption and remodeling of the healing bone, ultimately culminating in avascular necrosis [9, 26]. Until today, the literature is inconsistent regarding the question whether using high ITVs will increase EBL or lead to higher implant failure rates [4, 5, 9, 12, 13, 18, 26]. According to mathematical models, there is a linear correlation between ITV and bone compression [21]. Therefore, it is important to understand the implant design and how the forces on the bone-to-implant contact are distributed. In the presented study, the use of a finite element simulation with the herein used implants indicates that the highest pressure rates are occurring at the implant apex, whereas the crestal part of the implant distributes only minor forces to the surrounding bone, regardless of the insertion torque used (Figs. 6 and 7). This could explain why the EBL shows no statistically significant difference between high and regular torque group in the presented study.

The ITV and/or ISQ are values taken for clinically assessing the primary stability of a dental implant [3, 20, 22, 26]. ITV is measured at the time of implant placement as a single measurement. ISQ measurements can be repeated over time during the healing process and therefore can be used to monitor the bone healing [20, 26]. Several publications emphasized the importance of conducting repeated measurements, as implant stability tends to decrease during the healing process owing to resorptive phenomena in the healing bone (weakest point around week 3–4). The final stability is reached after 8–12 weeks [19, 21, 23]. The use of ITV as only measurement to define primary stability can be misleading, as a high ITV does not necessarily correlate with a high ISQ value. ITV is a measurement that defines rotational stability as caused by mechanical friction of the implant in the bone, whereas RFA analysis giving the ISQ values is measuring the lateral stiffness of the bone-to-implant interface and the stiffness of surrounding bone. In the presented study, there was only limited and moderate linear correlation between ITV and ISQ which is in accordance with existing literature [19, 26], whereas the bone density classes 1–4 according to Lekholm and Zarb [17] are correlating significantly both to ITV and ISQ. This again is in accordance with literature [3, 22, 28]. The correlation coefficient could potentially be higher if bone density had been assessed using an objective bone density assessment calculated from CBCTs [28], rather than the subjective assessment according to Lekholm and Zarb [17].

The categorisation of the bone qualities found in the clinical cases into classes 1–4 as described by Lekholm and Zarb [17] is a subjective clinically judgement of the surgeon. In the presented single center study, all surgical procedures were performed by the same skilled clinician. This eliminates a possible bias as would have been created by different clinicians with different clinical experience.

In current publications, a great variety of ITVs varying from < 10 to > 100 Ncm are described. Based on the systematic review by Lemos et al. [18] and Atieh et al. [2], the dividing criteria between high and regular torque groups was defined as 50 Ncm. To avoid possible damage either to the implant´s inner connection or to the bone by application of excessive force, a limit of 80 Ncm was set for all surgeries.

Other limitations are the retrospective character of the study and the imbalance of the study groups. However, the bone quality and associated insertion torque during surgery as patient individual parameters seem to be representative from experience.

## Conclusions

From the results of the presented study, it can be concluded that:

1. There was no statistically significant difference in marginal bone level changes between the „high torque“ and „regular torque“ group and very little bone loss was detected for this specific implant type.
2. No implant failure and no complications occurred in the observed study period.
3. In the presented clinical data, ISQ values and bone density are correlating. This is in accordance with recent literature.
4. In the presented clinical data, ITV and bone density are correlating. This is in accordance with recent literature.
5. RFA and ITV showed a weak to moderate correlation to each other. However, with ITV > 50 Ncm, there was no correlation anymore. This again reflects recent literature.

## Data Availability

The datasets used and/or analysed during the current study are available from the corresponding author on reasonable request.

## Abbreviations

BLC: Marginal bone level changes (mm)
EBL: Early bone loss
FEM: Finite element method
ISQ: Implant stability quotient
ITV: Insertion torque value (Ncm)
MBL: Marginal bone level loss
RFA: Resonance frequency analysis
SD: Standard deviation
